# Overlap syndrome of seronegative primary biliary cholangitis and small duct primary sclerosing cholangitis: a first case report and literature review

**DOI:** 10.2144/fsoa-2023-0187

**Published:** 2024-05-20

**Authors:** Salma Souissi, Sarah Laabidi, Nadia Ben Mustpha, Ines Chelly, Meriem Serghini, Monia Fekih, Asma Laabidi, Jalel Boubaker

**Affiliations:** 1 Departement of Gastroenterology A, La Rabta Hospital, Tunis, 1007, Tunisia; 2 Faculty of Medicine of Tunis, University of Tunis El Manar, Tunis, 1007, Tunisia; 3 Departement of Anatomopathology, La Rabta Hospital, Tunis, 1007, Tunisia

**Keywords:** overlap syndrome, primary biliary cholangitis, primary sclerosing cholangitis

## Abstract

Primary biliary cholangitis (PBC), primary sclerosing cholangitis (PSC), and autoimmune hepatitis (AIH) are distinct liver diseases. Cases combining PBC and PSC, are extremely rare. Here, we present a case of a 39-year-old woman with a history of colonic Crohn's disease treated with azathioprine. Discontinuation of the medication was prompted by abnormal liver function tests, but subsequent evaluations revealed persistent liver injury. Extensive diagnostic investigations, including imaging, serological tests, and liver biopsy, were conducted leading to a diagnosis of PBC-PSC overlap syndrome based on the presence of concentric lamellar fibrosis and chronic non-suppurative destructive cholangitis. The patient responded well to ursodeoxycholic acid treatment. This case emphasizes the importance of recognizing and diagnosing rare overlap syndromes, particularly those involving PBC and PSC, to ensure appropriate management and improve patient outcomes.

## Abstract (plain language summary)

Overlapping between PBC and PSC does exist.

Liver biopsy remains mandatory to recognize and better define this syndrome.

UDCA at normal dose seems to be an efficient therapeutic option.

Primary biliary cholangitis (PBC), primary sclerosing cholangitis (PSC), and autoimmune hepatitis (AIH) are liver diseases with increasing prevalence [1]. While they are considered separate entities, the coexistence of these diseases in the form of overlap syndromes is well documented. The diagnosis of these syndromes relies on a combination of clinical features, biochemical and serologic tests as well as radiographic and histological findings [2,3]. Among the various overlap syndromes, the most common associations involve AIH and PBC, followed by AIH and PSC, albeit with a lower prevalence. Reported cases of overlap syndrome involving PBC and PSC have been exceptionally rare, with only 13 instances documented thus far. Notably, none of these cases correspond to the combination of PSC and seronegative CBP [4]. The reason behind the infrequency of this particular combination compared with other overlap syndromes remains unclear. In this context, we present a case study of a 39-year-old woman with an overlap syndrome characterized by seronegative PBC and PSC.

## Case report

We present a case of a 39-year-old woman who has been under our department's care since 2012 for colonic Crohn's disease. The inflammatory bowel disease (IBD) had been effectively managed with salazopyrin until 2020 when she experienced a severe flare-up, requiring corticosteroid treatment. Subsequently, on 23 October 2020, azathioprine was prescribed as a maintenance therapy for her. During regular follow-up appointments, while monitoring response to azathioprine, biochemical tests revealed abnormal liver function: aspartate aminotransferase (AST) levels were elevated at 78 IU/l (normal range: <46), alanine aminotransferase (ALT) levels were elevated at 88 IU/l (normal range: <69), gamma-glutamyl transpeptidase (GGT), levels were significantly elevated at 320 IU/l (normal range: 12–43), alkaline phosphatase (ALP) levels were elevated at 423 U/l (normal range: 21–92), bilirubin levels were slightly elevated at 10 mg/l (normal range: <10), and albumin levels were within the normal range at 43 g/d (normal range: 35–53). However, blood count remained normal, and no physical signs were observed during the examination. Given the disturbance in liver function, azathioprine-induced hepatotoxicity was suspected, and the medication was immediately discontinued. Liver function tests were repeated on multiple occasions, consistently showing persistently elevated liver enzyme levels and signs of cholestasis, indicating ongoing liver injury or worsening of the condition.

**Figure 1.**
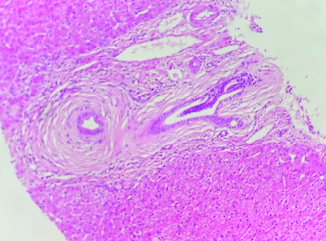
Concentric lamellar fibrosis around the bile ducts; hematoxylin-eosin ×40.

**Figure 2.**
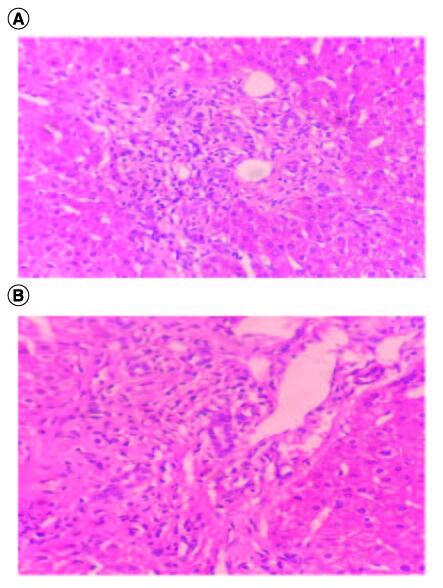
(A & B) Lymphocytic infiltration targeting the bile ducts associated to biliary neocanals with ductopenia; hematoxylin-eosin ×100.

The ultrasound examination ruled out any morphological signs of advanced liver disease or abnormalities in the biliary tract. A viral serological screen yielded negative results for hepatitis A virus, hepatitis B virus, hepatitis C virus, human immunodeficiency virus, cytomegalovirus and Epstein–Barr virus. There were no indications of insulin resistance (Homa index: 2.2) or underlying liver storage disorders such as iron, copper, or alpha-1 antitrypsin abnormalities. The autoimmune profile, assessed through indirect immunofluorescence, yielded normal results. Specifically, tests for anti-nuclear (ANA), anti-mitochondrial (AMA), and anti-muscle antibodies (SMA V pattern) were negative, as were the SP-100 and GP-210 antibodies. Other autoimmune tests also produced negative results. The levels of immunoglobulins were within the normal range. To further investigate, a MRCP was performed, which revealed no abnormalities. The intrahepatic bile ducts appeared regular without any strictures. Furthermore, a pharmacovigilance investigation was conducted by the pharmacology team of our hospital, which ruled out azathioprine as the cause of these abnormalities. As the laboratory tests consistently showed abnormal results, a liver biopsy was performed to determine the underlying cause of the cholestasis and cytolysis. The liver tissue specimen exhibited concentric lamellar fibrosis around some of small interlobular bile ducts ‘onion skin fibrosis’ corresponding to sclerosing cholangitis (Figure 1). There was no evidence of interface hepatitis. Lymphocytic infiltrates attacking the bile ducts were observed in several portal spaces additionally to the presence of biliary neocanals with a ductopenia. Cholangitis lesions were also observed (Figure 2). Based on these findings, the pathologist diagnosed the patient with PBC-PSC overlap syndrome. Subsequently, the patient was prescribed ursodeoxycholic acid (UDCA) at a dosage of 15–20 mg/kg/day, resulting in a favorable clinical and biochemical response.

## Discussion

Overlap syndrome encompasses various autoimmune hepatobiliary diseases, including AIH, PBC, PSC and AIC, coexisting within a single individual. These conditions exhibit a combination of hepatitic and cholestatic biochemical and histological features associated with AIH, PBC, PSC and/or AIC. Without appropriate treatment, they often progress toward liver cirrhosis and liver failure [5]. The overlap between PBC/AIH, AMA-negative PBC/AIH, and PSC/AIH has been documented in different studies [^6-8^]. However, there have been only a few reported cases of overlap between PBC and PSC [4]. Since both PBC and PSC are cholestatic diseases characterized by elevated levels of ALP and GGT, the coexistence of these two conditions can sometimes go unnoticed. Currently, no specific criteria exist for the diagnosis of PBC-PSC overlap syndrome, and it is typically identified when a patient meets the diagnostic criteria for both disorders [9].

In fact, both PSC and PBC contribute to common symptoms like jaundice, pruritus and fatigue, all attributed to cholestasis. However, most patients are asymptomatic in the early course of the disease [10].

The diagnosis of PBC can be established if two of three objective criteria are present: unexplained elevated ALP ≥1.5-times the upper normal value for over 24 weeks, serum AMA at titers ≥1:40 or if AMA negative, positivity of anti-sp100/anti-gp 210 and compatible liver histology, specifically non-suppurative cholangitis and interlobular bile duct injury. Elevated immunoglobulin M (IgM) levels are also seen [11].

Regarding PSC, its diagnosis involves identifying elevated serum markers of cholestasis (ALP, GGT) along with imaging results (MRCP or ERCP) that reveal distinct bile duct changes, including multifocal strictures and segmental dilatations, when all other possible cholestatic disorders and secondary causes are excluded. In biopsy analysis, PSC presents ‘classically’ with fibrous obliteration of small bile ducts, with concentric replacement by connective tissue in an ‘onion skin’ pattern. Although this histology is found in less than 25% of liver biopsies, it supports the diagnosis of PSC when present [12].

Indeed, in contrast to the widely accepted diagnostic criteria for autoimmune liver diseases there is still a lack of well-defined, validated and internationally agreed upon criteria for these variants especially when co-existing. Thus, the diagnosis of overlapping PBC/PSC, or PBC or PSC with coexistent AIH, remain difficult unless the clinical, radiological and histopathological findings are put together.

The patient described in this report presented with an unremarkable clinical picture, initially showing biochemical evidence of cholestatic disease, which could be indicative of both PBC and PSC. The initial serologic investigation did not provide a specific diagnosis. However, due to the persistent abnormal laboratory findings, further diagnostic procedures were performed. MRCP showed no abnormalities, and a liver biopsy was conducted, leading to the diagnosis of overlapping PBC/PSC syndrome.

A review of the literature revealed 13 reported cases of overlapping PBC/PSC. The first case was reported in 1984 by Rubel *et al.* The initial diagnosis of PBC was established through a combination of positive AMA, elevated ALP levels, and liver histology indicating portal fibrosis, bridging necrosis and the absence of periductal fibrosis [13]. Several attempts were made to visualize the extrahepatic biliary ducts through various procedures, but it was only after a percutaneous transhepatic cholangiogram (PTC) performed 7 years after the PBC diagnosis that multiple regions of stricturing and dilations, characteristic of the ‘beaded’ pattern diagnostic for PSC, were observed [4].

In September 2001, Burak *et al.* [14] presented a case involving a 40-year-old female who had been experiencing nocturnal, low-grade fevers and right upper quadrant pain for 2 years. Abnormal liver enzyme tests indicated a potential liver issue, and an abdominal ultrasound revealed the presence of gallstones. Further examination through endoscopic retrograde cholangiopancreatography (ERCP) showed irregular narrowing and dilatation of the central intrahepatic and proximal extrahepatic ducts, suggestive of PSC. The patient was prescribed UDCA at a daily dosage of 750 mg. A liver biopsy was conducted, which showed enlarged portal tracts with fibrosis and an inflammatory infiltrate composed of lymphocytes, neutrophils, and eosinophils. Bile duct destruction and cholestasis were evident, consistent with a diagnosis of PBC. Although the initial AMA test was negative using immunofluorescence, subsequent immunoblotting revealed the presence of antibodies to mitochondrial antigens. The patient experienced recurrent episodes of ascending cholangitis, leading to a repeat liver biopsy and ERCP. The biopsy confirmed the presence of established cirrhosis and classical changes associated with PSC, including lymphocytic portal infiltrates and pronounced onion-skinning fibrosis around medium-sized ducts.

In 2005, Kingham *et al.* [15] described two cases of patients, among a cohort of over 260 patients monitored for more than two decades, who exhibited immunological, biochemical, and/or histological criteria indicative of primary biliary cirrhosis (PBC), an interesting phenomenon emerged. Despite meeting the diagnostic criteria for PBC, their MRCP results suggested PSC. A similar case was reported by Sundaram *et al.* in 2018 involving a 35-year-old woman [9]. Jeevagan *et al.* [16] reported also the case of a middle-aged woman, who had been dealing with primary biliary cirrhosis (PBC) for 17 years, was admitted to the hospital due to clinical and biochemical symptoms characteristic of cholestatic syndrome. Initially, a provisional diagnosis of worsening PBC was considered. However, this diagnosis was proven incorrect following a MRCP scan, which revealed a typical benign stricture and dilatation of the common bile duct with a characteristic beading appearance suggestive of PSC. To manage this condition, the patient underwent stent placement and was prescribed an increased dose of UDCA, which led to an improvement in both her symptoms and her biochemical profile. Additionally, Floreani *et al.* reported on two patients who were eventually diagnosed with PSC/PBC overlap syndrome [17]. In one case the overlap condition was associated with psoriatric arthritis. The patient had a history of PBC suspected due to abnormal cholestasis enzymes and MRCP imaging. Subsequent liver histology confirmed the diagnosis of PBC. However, as time passed, repeat MRCP conducted over the years revealed an unusual pattern: upstream dilation of the bile duct with sections of marked stenosis, which was consistent with PSC. This patient had previously received a diagnosis of an ANA titer of 1:640, displaying a speckled pattern, and had tested positive for AMA-M2 and anti-GP210. Consequently, she underwent a successful dual treatment approach involving UDCA and an anti-tumor necrosis factor-alpha agent. In the second case, the primary condition initially presented as AMA-negative PBC, progressing over time to end-stage liver disease. Later, the patient either developed AMA positivity or displayed changes in the biliary tree consistent with PSC. Furthermore, Mandolesi *et al.* reported a case involving a 66-year-old woman who had initially been diagnosed with PBC based on histological and immunological features. Subsequent MRCP revealed intrahepatic bile ducts with irregular profiles and slight concentric wall thickening, without a dominant stricture [18]. Initially, she was treated solely with a high dose of UDCA (20 mg/kg/day) and showed a positive clinical and biochemical response. However, a follow-up MRCP was conducted due to earlier findings of minimal irregularities in the intrahepatic biliary tree. This follow-up MRCP documented the development of a dominant stricture in the bile duct in the fourth liver segment, while the common bile duct remained normal. Thus, overlap syndrome was made.

T Del Ross *et al.* reported another case of PBC/PSC overlap syndrome in 51-year-old female affected with psoriatic arthritis. The patient had biological and histological features of PBC [19]. He was first treated by UDCA at 10 mg/Kg/day that was later increased to 15 mg/Kg/day; a partial decrease in GGT and ALP levels was observed. Subsequently, the patient underwent an MRCP, which revealed a significant reduction in the right segmental duct extending for 2.5 cm, accompanied by upstream dilatation. Moreover, pronounced stenosis of the biliary tree was noted in the 5th and 6th hepatic segment, a characteristic finding indicative of PSC. For his arthritis symptoms, Adalimumab was administrated and fortunately, this treatment led to clinical improvement in arthritis symptoms and also lowered cholestasis indices suggesting a healing of both cholangiopathies.

Furthermore, only one case reported by Benabderrahmane *et al.* was associated with an IBD [20]. It was a first case of an overlap syndrome PBC/PSC in a context of autoimmunity and hypovitaminosis D. The diagnosis of PBC was made in the presence of cholestasis and positivity of AMA M2. MRCP showed a disorganized rarefied aspect of the intrahepatic bile ducts with a succession of short stenosis-dilatations. Ileocolonoscopy showed Crohn's ileitis and other investigations concluded to several autoimmune diseases: Hashimoto's thyroiditis, scleroderma and Gougerot-Sjögren's syndrome. The limit of this study was the absence of histological diagnosis, as the liver biopsy was refuted by the patient.

However, unlike all cases above, Oliveira *et al.* [21] reported a single case of an overlap syndrome combining PBC and small duct PSC. A 48-year-old woman was referred to a hepatologist due to elevated liver enzyme levels. She tested positive for ANA with a rim-like membranous and cytoplasmic speckled pattern, which raised suspicion of AMA positivity. A liver biopsy revealed several significant findings, including bridging portal fibrosis, a lympho-mononuclear infiltrate with lymphocytic interface hepatitis, and marginal ductular reaction. In some of the portal tracts, there were small biliary ducts displaying concentric fibrosis, resembling an ‘onion skin’ pattern, with duct obliteration. Subsequently, an MRCP was performed and the results were normal. Based on these findings, a diagnosis of small duct PSC was made, and she was treated with UDCA. Within 4 months of treatment, her liver enzyme levels progressively normalized.

In our case, the diagnosis was retained based on mainly histological criteria. In fact, the typical sign of PSC, consisting of concentric lamellar fibrosis or ‘onion skinning’ was found as illustrated in Figure 1 . In fact, this fibro-obliterative lesion represents the distinctive histological feature of PSC, it initiates as concentric rings of fibrosis, commonly referred to as onion-skinning, forming around bile ducts. Over time, this concentric fibrosis gradually constricts and eliminates the lumen of the bile duct, resulting in the formation of a fibrous plug or scar [22].

As for PBC, our histological examination found lymphocytic infiltrates attacking the bile ducts observed in several portal spaces additionally to the presence of biliary neocanals with a ductopenia. Cholangitis lesions were also observed as shown in Figure 2A & B. Indeed, histopathologically, the pathology of PBC is exclusively located in the intrahepatic small- or middle-sized bile ducts and is identified by the degeneration and necrosis of intrahepatic biliary epithelial cells, encircled by a concentrated infiltration of mononuclear cells known as chronic non-suppurative destructive cholangitis. This process induces destructive alterations, ultimately causing the disappearance of small- or medium-sized bile ducts [23]. It's important to notice that a subtle infiltration of lymphocytes commonly coincides with the fibrosis, although the level of inflammation is considerably less pronounced compared with PBC, as shown in our case.

As for the treatment of cholestatic liver diseases, UDCA is commonly utilized. UDCA primarily works by protecting cholangiocytes, stimulating hepatobiliary secretion, and safeguarding hepatocytes against apoptosis induced by bile acids [24]. The benefit of UDCA has been proven in PBC. However, in PSC, UDCA is responsible only for a biological improvement, without any effect on histological lesions [25]. As for overlap syndrome, there is currently no established standardized management for this disease. The strategy involves administering a higher dose of UDCA, typically ranging from 18 to 20 mg/kg/day.

In the current case, a complete biochemical response was observed, as indicated by normalized liver enzyme levels after one year of UDCA treatment. However, it should be noted that the patient was only monitored for a period of 2 years, and therefore, the long-term effectiveness and potential side effects of UDCA could not be reported.

## Conclusion

Overlap syndrome involving PBC and PSC is an exceptionally rare occurrence. After conducting a comprehensive literature review, we identified only 13 documented cases of PBC/PSC overlap syndrome. In the case of the patient discussed in this study, both MRCP and serological features appeared normal. Notably, this is the first reported instance of small duct PSC overlapping with seronegative PBC. Our findings highlight the importance of liver biopsy in accurately diagnosing this syndrome and contribute to a better understanding of this complex and debated topic.
